# Persistent high-risk behavior and escalating HIV, syphilis and hepatitis B incidences among men who have sex with men living in Bangui, Central African Republic

**DOI:** 10.11604/pamj.2018.29.132.12794

**Published:** 2018-02-23

**Authors:** Mbeko Simaleko Marcel, Longo Jean De Dieu, Camengo-Police Serge Magloire, Gérard Grésenguet, Ralph-Sydney Mboumba Bouassa, Danielle Piette, Beatrice Gulbis, Laurent Bélec

**Affiliations:** 1 Centre National de Référence des Infections Sexuellement Transmissibles et de la Thérapie Antirétrovirale, Bangui, Central African Republic; 2 Faculté des Sciences de la Santé de Bangui, Central African Republic; 3 Service de Gastroentérologie, Hôpital de l’Amitié, Bangui, Central African Republic; 4 Laboratoire de Virologie, Hôpital Européen Georges Pompidou and Université Paris Descartes, Bio Sorbonne Paris Cité, Paris, France; 5 Ecole de Santé Publique, Université Libre de Bruxelles, Bruxelles, Belgium

**Keywords:** Men having sex with men, HIV, syphilis, clinical care, intervention, Central African Republic

## Abstract

**Introduction:**

HIV in sub-Saharan Africa remains a great concern in men who have sex with men (MSM). Intervention on MSM is a key strategy to control the burden of HIV among this population. Herein we assessed the effect of 2 years of HIV testing and counseling on risk-tacking and HIV and STI incidences among MSM living in Bangui in the Central African Republic.

**Methods:**

The incidences of HIV, syphilis and hepatitis B and the sexual behavior characteristics were assessed at inclusion and after 2 years of follow up in the prospective MSM cohort.

**Results:**

99 MSM were included and followed up during 2 years. The mean age of study MSM was 24 years (range, 14-39); among those, the majority was single (84.8%) and unemployed (33.3%) or students (23.9%). The majority (up to 80%) were living in only 4 (out of 10) neighboring district of Bangui. Insertive anal intercourse showed significant decrease from 54% at inclusion to 46% after 2 years of follow up (P < 0.001). In contrast, we observed slight increase in receptive anal intercourse (60% versus 66%) and oral sex (70% versus 74%), but the difference did not reach statistical significance. Finally, the prevalences of HIV, syphilis and hepatitis B increased significantly from 29% to 41%, 12% to 21% and 14% to 23%, respectively.

**Conclusion:**

These observations indicate that medical care and counseling on MSM does not provide significant changes in risk-taking, whereas the incidences of HIV, syphilis and hepatitis B remained high. Innovative interventions should be conceived for the MSM population living in Bangui.

## Introduction

The burden of HIV epidemic in sub-Saharan Africa remains a great concern in high-risk populations such as men who have sex with men (MSM), who constitute a core group for HIV and several sexually transmitted infections (STI) such as syphilis and hepatitis B [1-9]. High-risk sexual behaviors such as unprotected anal intercourse and multiple sexual partners are the main factors closely associated with this high burden of HIV and STIs in this vulnerable population [10, 11]. Other factors such as social vulnerability, criminalization, stigma and lack of national intervention strategies for prevention and access to care for MSM are also recognized contributive factors in most of sub-Saharan African countries [12, 13]. Therefore, the implementation of interventions strategies for prevention and medical care towards the MSM population is a key strategy to control the burden of HIV infection and associated STIs in this vulnerable group [14, 15]. Indeed, through intervention strategies such as HIV and STIs testing and counseling, infected MSM can be identified and treated as early as possible. In other hand, promoting HIV counseling in MSM has proved to be an effective strategy to reduce HIV-related sexual risk behaviors, thereby decreasing the incidence of HIV infection among MSM [15-18]. In the Central African Republic, the HIV epidemic is generalized with a prevalence of 4.9% in adult population [19, 20]. Relatively little has been reported until now about MSM and their sexual health. One preliminary serosurvey conducted in 2010 on MSM in Bangui highlighted that MSM are an identifiable core group accumulating high-risk sexual behaviors and high prevalence of HIV (25%), hepatitis B (17%) and syphilis (4%) [20]. With 25%, the prevalence of HIV in MSM living in Bangui was about 5 times higher than the prevalence of HIV in general population. These data advocate the implementation of specific and targeted MSM interventions to reduce unsafe sexual practices, facilitate access to specialized health service for key vulnerable populations, thereby a priori rendering effective the reduction and control of HIV and associated STIs in this high-risk group [21-23]. Finally, in the present study, the incidences of HIV, syphilis and hepatitis B infection and the sexual behavior characteristics were assessed at inclusion and after 2 years of follow up in a prospective cohort of men who have sex with men (MSM) attending the Centre National de Référence des Infections Sexuellement Transmissibles et de la Thérapie Antirétrovirale (CNRIST/TAR), which constitutes the principal clinic for sexually transmitted infections (STI) of Bangui, the capital city of the Central African Republic.

## Methods

**Inclusion of study population and socio-demographic variables**: The CNRIST/TAR of Bangui includes specific care towards the MSM population of Bangui. Routinely, 170 MSM attend regularly the STI clinic for HIV and STI screening and care, to receive specific treatment, HIV counseling and for those positive for HIV global support. For the study purpose, a specific strategy involving peer educators was adopted in order to validate the inclusion of MSM attending the CNRIST/TAR and prevent bias. Thus, eligibility criteria were to be approved as having sex with men by his peers, to accept to be followed over a period of at least 2 years and to have a fully informed medical record. At inclusion, a standardized interview was conducted by a sociologist to collect socio-demographic characteristics, such as nationality, residency, age, religion, profession, marital status, number of sexual partners, number of children, age at first intercourse and frequency of condom use, sexual practices and finally to advise participants about HIV and associated STIs. This research study was reviewed and formally approved by Scientific Committee of the Faculté des Sciences de la Santé (“FACSS”) of Bangui (so-called “Comité Scientifique de Validation des Protocoles et des Résultats de Recherche en Santé”/ CSVPR), constituting the National Ethical Committee (Reference #2UB/FACSS/CSVPR/09) in Central African Republic. Informed written consent was obtained from all participants before enrollment. The collected data were anonymized before their analysis. Finally, a return of laboratory results to clinicians was conducted to achieve a better management of the treated patients.

**Figure 1:**
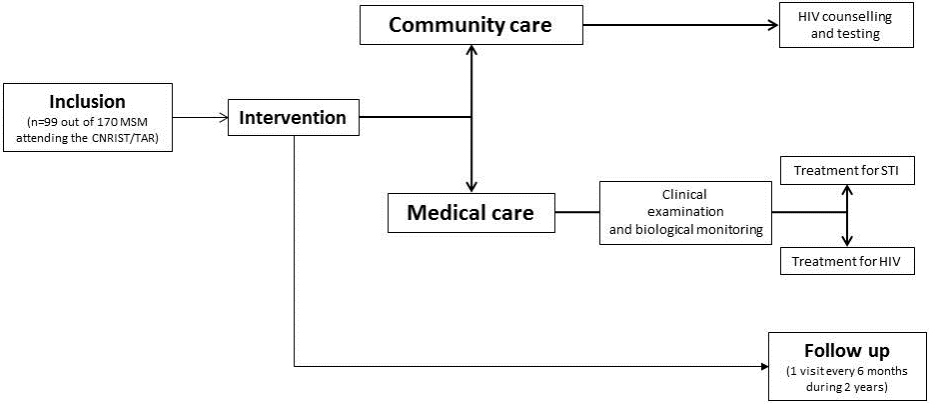
Flow chart showing the inclusion, the community care and medical care interventions and the 2 years follow up. The study population consisted in 99 men who have sex with men (MSM) included according to eligility criteria among 170 MSM regularly attending the Centre National de Référence des Infections Sexuellement Transmissibles et de la Thérapie Antirétrovirale (CNRIST/TAR) of Bangui

**Intervention and follow up**: One day after the interview, the follow-up of included MSM began with a medical appointment including clinical examinations and biological investigations for the diagnosis of the most common STIs including HIV, syphilis and hepatitis B (Figure 1). Biological results were returned 72 hours after and those positive for STIs received adapted treatment. The HIV-positive MSM were enrolled in the HIV cohort followed in the CNRIST/TAR. In order to retain the study participants, included MSM were asked to regularly come back to the medical center every six months for a biological checkup and to receive treatments, condoms and sexual behavioral counseling. The package of intervention consisted in HIV/STI counseling and condom distribution, clinical examination and biological monitoring and medical care for patients infected with STIs and HIV-infected patients. For HIV/STI counseling and condom distribution, a 7 to 10-minute interactive conversation on HIV, STIs, their modes of transmission and effective prevention way with the emphasis on condom use as an easy and effective prevention tool, was carried out by a sociologist towards study participants; at the end, the sociologist distributed to the participants as many condoms as required. A physical and clinical examination was carried out by a medical professional to investigate patients for symptoms of potential diseases. For laboratory testing, plasma or serum samples from blood collection by venipuncture in each patient were used for serological testing of HIV, syphilis and hepatitis B infection. HIV-positive status was determined by the national serological testing algorithm using Genscreen ULTRA Combo HIV Ag/Ab test (Bio-Rad Laboratories, Washington, USA), an enzyme immunoassay kit for the simultaneous detection of HIV p24 antigen and antibodies to HV-1 (group M and O) and HIV-2 in serum or plasma and the positive tests were confirmed by an enzyme-linked immunosorbent assay [ELISA] (Vironostika^®^ HIV Uni-Form II Ag/Ab, bioMérieux Marcy l'Etoile, France). Rapid plasma reagin non-treponemal test (RPR, Carbon, Cypress Diagnostics, Belgium) was used to detect reaginic antibody and positive tests were confirmed using the Treponema Pallidum Hemagglutinations Assay (TPHA, Syphagen, Biokit, Barcelona, Spain) a highly specific test to detect trepanoma antibody and then diagnose active syphilis. Finally, a Monolisa Enzyme Immunoassay HBsAg ULTRA (Bio-Rad Laboratories, Washington, USA) was used for the one-step determination of hepatitis B surface antigen (HBsAg) in the human plasma or serum. For medical care for patients infected with STIs and HIV-infected patients, HIV positive patients were included in the HIV cohort followed in the medical center and received antiretroviral treatment accordingly to the World Health Organization (WHO) consolidated guidelines on the use of antiretroviral drugs for treating and preventing HIV infection (WHO, 2016). Patients positive for one of the others STIs were regularly medically followed and received appropriate treatment.

**Outcome variables and statistical analysis**: A standardized form was used to collect information on demographic characteristics, sexual behaviors and STIs/HIV test result at inclusion and at the of the 2 years period of follow up. The effects of intervention towards the MSM population at CNRIST/TAR were assessed from two main outcomes variables: changes in sexual behaviors and the frequency of STIs/HIV. The first outcomes variable ("changes in sexual behavior") was estimated from behavioral variables such as the “sexual partner numbers in the last three months” and the “frequency of condom use with the last sexual partner” in the three categorical variables “never use condom”, “occasionally (irregularly) condom use” and “systematically (consistent) use condom”. The second outcomes variable included the prevalences of STIs/HIV. Stata 12 software (Science Plus Group, Groningen, The Netherlands) was used for statistical analysis. Descriptive analyses were performed by Pearson's chi-square test or Fisher's exact test and Mann-Whitney non-parametric U-test to compare categorical and non-categorical variable respectively.

## Results

**Study population**: A total of 99 MSM were included (Figure 1). The Table 1 depicts the principal socio-demographic characteristics of study population. Their mean age was 24 years (range, 14-39); among those, the majority was single (84.8%), whereas the remaining were living in couple with a sexual mal partner; the majority were unemployed (33.3%) or students (23.9%). The majority (up to 80%) were living in only 4 (out of 10) neighboring district of the capital city.

**Table 1:** Socio-demographic characteristics at inclusion of the 99 men who have sex with men (MSM) attending the Centre National de Référence des Infections Sexuellement Transmissibles et de la Thérapie Antirétrovirale (CNRIST/TAR), which constitutes the principal clinic for sexually transmitted infections of Bangui

Variables	N (%)	*P*
**Age group** (years)		
14-19	10 (10.1)	< 0.001
20-24	51 (51.5)	
25-29	21 (21.2)	
30-34	14 (14.1)	
35-39	3 (3.1)	
**Marital status**		
Life couple (with male partner)	15 (15.4)	0.51
Single	84 (84.8)	
**Professional activity**		
Civil servant	8 (8.3)	0.002
Student	23 (23.9)	
Merchant	11 (11.5)	
Unemployed	32 (33.3)	
seller	22 (22.9)	
**Living place**		
District 8	26 (26.3)	0.08
District 4	15 (15.2)	
District 5	18 (18.2)	
District 10	10 (10.1)	
District 3	13 (13.1)	
District 2	8 (8.1)	
District 6	5 (5.1)	
District 1	2 (2.1)	
District 9	2 (2.1)	

**Figure 2:**
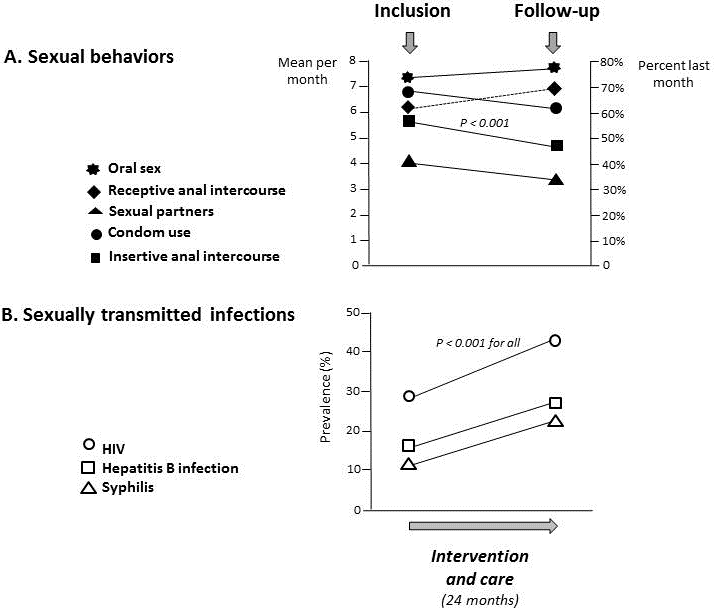
Changes in sexual behavior (number of sexual partners: mean per month; receptive anal intercourse, practice of oral sex, condom use and insertive anal intercourse: percent last month) and principal sexually transmitted infections, including HIV, syphilis and hepatitis B infection (prevalences of laboratory diagnosis) in the study MSM from inclusion to the end of 2 years follow up

**Effect of medical care and HIV counseling**: The changes in sexual behavior and principal STIs during the study period are depicted in the Figure 2. Concerning the changes in sexual behavior, the interviews conducted during the inclusion and medical visits of the MSM from inclusion to the end of 2 years follow up revealed that only the frequency of insertive anal intercourse was significantly reduced from 54% to 46% (P < 0.001) (Figure 2, A). There was no statistically significant variation in the frequency of condom use, oral sex or in the mean number of sexual partners between 2010 and 2012 (Figure 2, A). In addition, all other high-risk sexual behavior increased slightly but without significant difference during the study period: receptive anal intercourse (60% versus 66%) and oral sex (70% versus 74%). The mean number of sexual partners showed a trend to decrease from 4.0 ± 3.0 per month at inclusion to 3.0 ± 3.1 after 2 years, but the variation was not significant (P = 0.20). Furthermore, there was a slight decrease in condom use with the last sexual partner during the study follow up (68% versus 60%), without significant difference. Concerning the changes in STIs frequencies, the variations of the prevalences of HIV, syphilis and hepatitis B infection were evaluated in the 99 study MSM from inclusion to the end of 2 years follow up (Figure 2, B). The prevalences of HIV, syphilis and hepatitis B infection increased between the study period from 29% to 41%, 12% to 21% and 14% to 23%, respectively (P < 0.001).

## Discussion

In the present study, we report for the first time in MSM living in the Central African Republic, a very high prevalence of HIV infection ranging from 20% to 41%, as well as syphilis, ranging from 12% to 21%. Our observations also point that medical care and counseling on sexual risk behavior as currently carried out in the CNRIST/TAR, the principal STI clinic center in Bangui, in the direction of the MSM population does not provide significant changes in MSM sexual practices and risk-taking. In contrast, high risk sexual practices, including the very high risk receptive anal intercourse, showed no significant variations during the study period, although insertive anal intercourse decreased significantly during the intervention and the incidences of HIV, syphilis and hepatitis B infection remained high. To our knowledge, our study focused on Bangui's MSM is unique in the field of HIV epidemic in the Central African Republic, where available epidemiological data on HIV infection were restricted until now only to heterosexuals or other vulnerable populations such as HIV-infected children [24] and female sex workers [25]. These findings highlight the difficulties and pitfalls of changing high-risk sexual behavior among the Bangui's MSM population, and emphasize the need to design innovative interventions in the direction of this particularly vulnerable population living in the Central African Republic. High prevalences of HIV, syphilis and hepatitis B infection were observed in study MSM population. These observations are similar to findings reported in MSM living in sub-Saharan African. Thus, studies conducted in sub-Saharan African countries in the last 10 years show that HIV prevalence among MSM is more than 5-18 times higher as compared to general population [2]. Thus, in Tanzania the prevalence of HIV in MSM was 17.4% and 3.7% in the general population [9]. Similarly, in Malawi, the prevalence of HIV was 21.4% in MSM and 6.1% in the general population [1]. In Kenya, the HIV prevalences in MSM ranged from 12.3% to 43.0% as compared to 6.1% in the general population [5]. In Ivory Coast, the prevalence of HIV in MSM was as high as 50% (n=96) as compared to 3.2% in the general population [3]. Similarly, high levels of syphilis and hepatitis B have also been frequently reported in MSM living in sub-Saharan Africa, especially in HIV-infected individuals [7].

MSM in Bangui are likely more numerous as previously thought. Our study show that they are living restricted to a limited number of neighboring highly populated districts of the capital city. They are single and unemployed or students. These observations are consistent with the socio-demographic situation of MSM described in numerous sub-Saharan African countries, such as for example in Mozambique and Malawi [26, 27]. In our present series, one to ten (around 10%) MSM were very young, less than 19 year old. Early sexual debut is considered as a major risk factor for acquiring HIV infection and STI in young African men. Thus, MSM living in Kenya, who had sexual debut between 16 and 21 years were 1.8 times more likely to be infected with HIV, as compared with those who never had sex [28, 29]. Young men early starting sexual intercourse, especially before the age of 16 years, are facing psychological problems related to drugs and may be at risk of suicide [28]. Although the stigma and criminalization of MSM population in many sub-Saharan African countries forces these populations to remain hide, this vulnerable group at high risk for HIV infection and associated STI appears well present in sub-Saharan countries and needs to be taken into account in order to control the HIV epidemic. Thus, it is well admitted that behavior change interventions can reduce unsafe sexual practices [15-18]. Indeed, counseling before and after HIV testing is not only a critical entry point for biomedical HIV prevention interventions, such as pre-exposure prophylaxis and early treatment initiation, but is also an opportunity for focused risk reduction adapted counseling targeting every individual according to their life circumstances and it can therefore contribute to behavior change and reducing risk-taking [30]. In our study, we thought that mobilizing MSM in the principal STI clinical center of Bangui, providing counseling and HIV testing, as well as diagnosing and treating common STIs, could help to improve the frequency of condom use, reducing the number of sexual partners and finally reduce the burden of HIV and associated STIs. However, our study reporting two years of prospective follow up demonstrates that the currently proposed actions in the direction of the MSM population produced only marginal effects and are globally insufficient.

Our observations suggest that earlier interventions could have a major impact towards fighting HIV and STIs in young MSM in the Central African Republic. However, after 24 months of medical and community counseling interventions, although the frequency of sexual intercourse (insertive anal intercourse) decreased significantly, no significant changes in other high risk sexual practices could be observed. Only a small number of participants had reduced the number of their sexual partners during the 2 years intervention period and only a marginal number of MSM declared having always used condom during sexual intercourse. Moreover, the prevalences of HIV, syphilis and hepatitis B infection increased significantly between the study period from 29% to 41%, 12% to 21% and 14% to 23%, respectively. These findings are consistent with a previous report in MSM in China by Lau and colleagues, showing that HIV and STI testing and counseling did not significantly reduce the prevalence of HIV and sexual risk behaviors in MSM [31]. In Lau's study, HIV prevalence ranged from 2.5% at baseline to 6.3% after 21 months of intervention and participants interviewed at month 21 self-reported increased in unprotected anal intercourse and other risk behaviors, comparing recent and pre-baseline experiences [31]. However, in another Chinese study, it was shown that after 3 months of HIV and STI testing and risk reduction counseling, anal intercourses decrease significantly among STI-infected MSM from 73.1% to 38.5%, group sex decrease from 19.2% to 5.8% and unprotected anal intercourse decrease from 23.1% to 5.8% [32]. Among STI-negative MSM, risk sexual behaviors could also decrease significantly [32]. Likewise, Huan and colleagues reported in China significant decrease in both unprotected anal intercourse from 60.9% to 42.9% and in the number of MSM having sex with several partners, following 1.5 years intervention [33]. The possibility that relapse in high risk sexual behavior could have occurred in our study MSM should be further investigated, as suggested by previous studies showing that significant positive effects of intervention may wane over time [22, 33, 34].

## Conclusion

In conclusion, MSM in the Central African Republic constitute a key population with high prevalence of HIV and STI. Because of their high-risk sexual behaviors, the control of the HIV epidemic in this group appears very difficult to achieve even by applying classical strategic interventions such as HIV testing and risk reduction counseling that have already proven to be effective in other contexts. It would be useful to associate basic prevention activities using more active pedagogical methods such as groups of interviews, peer education, motivational interviews or counseling based on a structured educational approach and provide support for HIV patients with antiretroviral drugs.

### What is known about this topic

- In sub-Saharan Africa, MSM constitute a core group for HIV and several sexually transmitted infections such as syphilis and hepatitis B;
- High-risk sexual behaviors such as unprotected anal intercourse and multiple sexual partners are the main factors closely associated the high burden of HIV and STIs in MSM;
- HIV and STIs testing and counseling is a key strategy to reduce sexual risk-taking and control the burden of HIV infection and associated STIs in MSM.

### What this study adds

- There is a very high prevalence of HIV infection as well as syphilis in MSM living in Central African Republic;
- Medical care and counseling on sexual risk behavior as currently carried out in the CNRIST/TAR, the principal STI clinic center in Bangui; in the direction of the MSM population does not provide significant changes in MSM sexual practices and risk-taking;
- There is an urgent need to design innovative HIV prevention interventions in the direction of MSM population living in the Central African Republic.
